# DNA Mini-Barcoding: A Derived Barcoding Method for Herbal Molecular Identification

**DOI:** 10.3389/fpls.2019.00987

**Published:** 2019-08-28

**Authors:** Zitong Gao, Yang Liu, Xiaoyue Wang, Xuemin Wei, Jianping Han

**Affiliations:** Chinese Academy of Medical Sciences & Peking Union Medical College, Institute of Medicinal Plant Development, Beijing, China

**Keywords:** DNA mini-barcoding, DNA barcoding, molecular identification, natural herbal products, biomarker

## Abstract

In recent years, the demand for natural herbal products (NHP) has increased; however, the quality of these products is difficult to confirm due to the lack of a comprehensive quality control system. Traditional methods are not effective in detecting processed ingredients. DNA barcoding is an established technique that has been used for more than 10 years. This technique uses short standard sequences (generally 200–600 bp) to identify species. While a complete DNA barcode is difficult to obtain from NHP due to DNA degradation, mini-barcoding is a complementary tool to identify species in NHP. DNA mini-barcoding uses smaller DNA segments for polymerase chain reaction amplification and can be applied to identify species rapidly. The present review summarizes the development and application of DNA mini-barcodes over recent years and discusses the limitations of this technique. This review also compares mini-barcoding and meta-barcoding, a technique using universal polymerase chain reaction primers to simultaneously amplify multiple DNA barcodes and identify many species in a single environmental sample. Additionally, other detection methods that can be combined with mini-barcodes, such as nucleotide signatures, high-resolution DNA melting analysis, and gold nanoparticles, are discussed. DNA mini-barcoding can fill the gaps left by other methods in the field of herbal molecular identification.

## Introduction

Natural herbal products (NHP) use herbs as raw materials and include herbal medicines, dietary supplements, and herbal extracts. In recent years, worldwide demand for NHP has increased, making NHP adulteration and counterfeiting a global problem (Fotiou et al., 2009; Mackey and Liang, 2013; Gaudiano et al., 2016). Approximately 10% of medicines in developing countries are counterfeit, and this problem is particularly pronounced in Africa, Latin America, and Asia (Medina et al., 2016; Shanmughanandhan et al., 2016). In developed countries, counterfeit drugs also circulate in illegal supply chains (Gaudiano et al., 2016). In addition, primary herbal ingredients may be adulterated, typically by substitution with cheaper, off-label fillers. These unlabeled substitutions undoubtedly reduce therapeutic effectiveness and pose serious risks to consumer health (Newmaster et al., 2013; Xin et al., 2015; Han et al., 2016; Mishra et al., 2016). For example, Newmaster et al. found unlabeled ingredients in 59% of Chinese herbal medicine products in North America, with nearly one-third of verifiable products contaminated or supplemented, and only two samples that matched their labels (Newmaster et al., 2013).

The currently available NHP chemical detection methods are not sufficient (Raclariu et al., 2018). These methods include thin-layer chromatography, mass spectrometry, high-performance liquid chromatography (Li et al., 2011), and nuclear magnetic resonance metabolomics (Booker et al., 2016). Chemical variation in different batches of NHP depends on geographic location, storage conditions, and processing methods (Parveen et al., 2016), posing difficulties for proper chemical analyses and objective judgment (de Boer et al., 2015). In addition, although chemical detection markers are primarily selected from representative chemical compounds within the tested species, they may not be species-specific. Thus, chemical methods cannot distinguish species that share chemical markers, possibly misidentifying the source of NHP raw materials (Poornima, 2010). In recent years, NHP safety issues have received considerable attention (Zhang et al., 2012; Palhares et al., 2015; Pruitt et al., 2018; Liu et al., 2018a). More effective detection methods are urgently required for more comprehensive NHP identification.

Molecular markers are widely used to identify species accurately and rapidly. The stability of DNA provides an advantage over other macromolecules, such as RNA and protein (Mishra et al., 2016). Among DNA markers, DNA barcoding is considered the best tool for species taxonomy and identification. DNA barcoding is an established technique using short standard DNA sequences as markers for species identification. DNA can be extracted from nuclei, chloroplasts, or mitochondria (<1,000 bp) (Hebert et al., 2003) and is typically derived from six major candidate regions, including maturase K (*matK*), photosystem II protein D1-tRNA-His, ribulose-1,5-bisphosphate carboxylase/oxygenase large subunit (*rbcL*), internal transcribed spacer (ITS), internal transcribed spacer 2 (ITS2), and cytochrome c oxidase I (CO1) (Techen et al., 2014). DNA barcoding does not require any obvious morphological characteristics or specialized training, and is compatible with standard databases (Chen et al., 2014). DNA barcoding generally requires DNA extraction, polymerase chain reaction (PCR), sequencing, and sequence analysis. In recent years, this approach has been widely tested and validated for the identification of medicinal plants (Chen et al., 2010; Techen et al., 2014; Mishra et al., 2016).

DNA barcoding has been successful in the efficient and inexpensive identification of herbal materials; however, it is not suitable for identifying processed NHP (Parveen et al., 2016). DNA degradation may occur during the production of NHP and can significantly decrease the efficiency of PCR. Also, some additives mixed into NHP can cause PCR failure. Moreover, commonly used DNA barcode sequences are not suitable for distinguishing individual species due to primer uniformity. The deficiencies of DNA barcoding have been indicated in various studies (Bauer et al., 2003; Novak et al., 2007).

DNA mini-barcoding, using a smaller length of DNA compared with traditional barcoding, may help overcome the difficulties associated with DNA barcoding. (Meusnier et al., 2008). Mini-barcodes, generally ≤200 bp, can be amplified more rapidly than regular barcodes, owing to their shorter size (Meusnier et al., 2008; Srirama et al., 2014). Unlike traditional DNA barcodes, mini-barcodes are more diversified and are able to distinguish between limited species. Based on specially designed primers, mini-barcodes can accurately identify targeted species.

The present review emphasizes the utility of DNA mini-barcoding, summarizes key technologies utilized in this field, and discusses deficiencies associated with this technique. Furthermore, this review outlines technologies that could be developed in combination with mini-barcoding to improve NHP identification.

## DNA Mini-Barcoding for Natural Herbal Products Identification

NHP often undergoes complex processing, including pulverization, extraction, leaching, purification, concentration, drying, and granulation. For example, the production of Shuanghuanglian particles involves boiling Lonicerae japonicae Flos for 1.5 h, batch filtering, and concentration (Chinese Pharmacopoeia Commission, 2015). As this type of processing can destroy the DNA structure, it is difficult to obtain complete target barcodes, hampering successful species identification.

DNA mini-barcoding has developed extensively over the previous 10 years. Many research studies have explicitly highlighted the importance of DNA mini-barcoding as an extension of DNA barcoding. In 2007, Taberlet et al. demonstrated that a short region of tRNA-Leu (*trnL*) in chloroplasts, termed P6 loop (10–143 bp), could be amplified from processed food and permafrost samples (Taberlet et al., 2007). In 2008, Meusnier et al. proposed mini-barcodes as a way to overcome difficulties associated with amplifying degraded DNA. Analyzing 100 and 250 bp of DNA in the CO1 region achieved successful identification rates of 90 and 95%, respectively. This study also developed universal primer pairs for mini-barcodes of 120–150 bp, and achieved higher success rates for these amplicons versus full-length barcoding (Meusnier et al., 2008). Sarkinen et al. found that shorter amplicons were linked to higher PCR success rates (Sarkinen et al., 2012). Little selected 12 DNA mini-barcodes from *rbcL* and PCR amplification for all mini-barcodes was successful for 90.2–99.8% of species estimated by validated electronic simulation (Little, 2014).

DNA mini-barcodes have broadened the utility of DNA barcoding and are suitable to assess NHP quality. For instance, Cheng et al. extracted DNA from six samples of the herbal traditional Chinese medicine (TCM) Liuwei Dihuang Wan, amplified the *trnL* and ITS2 regions, and subjected them to Sanger sequencing. Owing to DNA damage, traditional barcoding could not be used to analyze the DNA (Cheng et al., 2014). Lo et al. illustrated that after TCMs were boiled for 120 min, 88-bp DNA fragments could be successfully amplified, whereas 121-bp DNA fragments were not amplified (Lo et al., 2015). Song and colleagues showed a PCR success rate of 8.89–20% for processed medicinal materials (Song et al., 2017). Table 1 summarizes recent studies on NHP, including dietary supplements, Chinese patent medicines (CPMs), TCM decoctions, and herbal infusions, based on DNA mini-barcoding over the previous 5 years. These studies demonstrate the utility of DNA mini-barcoding for NHP identification.

**Table 1:** Overview of DNA mini-barcode applied in the identification of natural health product in recent 5 years.

NHPs	Ingredients (plants in Latin name)	DNA mini-barcode	Methods	References
Concentrated Chinese medicine granules	*Angelica sinensis, Panax notoginseng*	ITS2	DNA extraction, adaptor ligation-mediated PCR	Lo and Shaw, 2018b
*Ginkgo biloba* herbal products	*Sophora japonica*	Short region in ITS2	DNA extraction, PCR with specific primer pairs, sequencing	Liu et al., 2018b
*Ginkgo biloba* herbal products	*Ginkgo biloba*	Short region in *rbcL*	DNA extraction, Recombinase polymerase amplification with lateral flow strips(RPA-LFS)	Liu et al., 2018b
Decoction pieces	Catechu, Arecae Pericarpium, Asparagi Radix, Gastrodiae Rhizoma, Bletillae Rhizoma, Paeoniae Radix Alba, Curcumae Longae Rhizoma, Chaenomelis Fructus, Ginseng Radix Et Rhizoma Rubra, Bolbostemmatis Rhizoma, Rehmanniae Radix Praeparata, Nelumbinis Rhizoma Nodus, Zingiberis Rhizoma, Schisandrae Chinensis Fructus, Sparganii Rhizoma	P6 loop of *trnL*(UAA)intron region	Processing method recorded in Chinese pharmacopoeia, DNA extraction, PCR, cloning and sequencing	Song et al., 2017
Chinese patent medicines	Lonicerae japonicae Flos	Short region in ITS2	DNA extraction, PCR with specific primer pairs, sequencing, SNP sites analysis	Gao et al., 2017
Chinese patent medicines	Eucommiae Folium	Short region in ITS2	DNA extraction, PCR with specific primer pairs, electrophoresis	Gao et al., 2017
Fang Feng product	*Saposhnikovia divaricata*	ITS2	DNA extraction, PCR, sequencing, structure analyze	Zhang et al., 2016
Chinese patent medicines	Angelicae Sinensis Radix	Short region in ITS2	DNA extraction, PCR with specific primer pairs and sequencing	Wang et al., 2016
Chinese patent medicines	*Panax quinquefolius*	Short region in ITS2	DNA extraction, PCR with specific primer pairs and SNP sites analysis	Liu et al., 2016
Herbal infusions	Hypericum perforatum, Hypericum androsaemum	Short region in ITS1 and *matK*	DNA extraction, Qualitative PCR Real-time PCR amplification with specific primer pairs, HRM analysis	Costa et al., 2016
Capsules, tablets	*Phyllanthus amarus*	*trnL*	DNA extraction, PCR amplification, sequencing and primer design, Determination of primer specificity, HRM analysis	Buddhachat et al., 2015
TCMs of ginsengs	*Panax notoginseng, Panax ginseng*	*ycf1a*, *ycf1b*	Chloroplast genome sequencing, Genome annotation, Identification of the hypervariable regions, Plant material, PCR amplification and hypervariable region sequencing	Dong et al., 2014
Herbal dietary supplements	*Ginkgo biloba*	*matK*	DNA extraction, PCR and sequencing	Little, 2014
Saw palmetto herbal dietary supplements	*Serenoa repens*	Short region in *rbcL, matK*	DNA extraction, PCR and sequencing	Little and Jeanson, 2013

## The Challenge of DNA Extraction and Improved Methods for Natural Herbal Products

In the cells of medicinal plants, secondary metabolites including fibers, proteins, polysaccharides (Moreira and Oliveira, 2011), parenchyma, alkaloids, and flavonoids all covalently combined with double-stranded DNA molecules (Sahu et al., 2012). During processing, artificial pigments, starch, salt, saccharin, tannins, fatty acids (Opel et al., 2010; Schrader et al., 2012; Newmaster et al., 2013), oils, or wax are often added to NHP as preservatives or to improve taste. Both secondary metabolites and added ingredients reduce the quality of extracted DNA and inhibit PCR amplification (Madesis et al., 2014; Lo and Shaw, 2018a).

Low-quality DNA is difficult to amplify and sequence; thus, DNA quality greatly influences the identification efficiency of the mini-barcoding method (de Boer et al., 2015). Chen et al. found that extracted DNA was viscous and opaque when CPM DNA extraction was performed without a column DNA purification kit to remove impurities, and that amplification products could not be obtained from unpurified DNA in this case (Chen et al., 2012). Additional research revealed that the amplification success rate of the barcoding high-resolution melting analysis (Bar-HRM) depended on high-quality DNA. Non-specific PCR products were produced from low-quality DNA templates, resulting in incorrect genotyping and low sensitivity (Mishra et al., 2016).

Extraction of high-quality DNA from NHP is the initial step and is critical to successfully apply molecular diagnostics (Rohland and Hofreiter, 2007; Telle and Thines, 2008). Conventional DNA extraction methods include the cetyltrimethylammonium bromide (CTAB) method, magnetic bead kits, and the sodium dodecyl sulfate method. However, these methods must be modified because of different secondary metabolites in CPMs. DNA extraction methods have been improved by facilitating cell lysis, extending preparation time, and increasing the number of steps in DNA purification and concentration. Chen et al. used a commercial DNA purification kit to extract high-quality DNA from oral liquids and injections (Chen et al., 2012). Cheng et al. improved DNA precipitation by using 70% methanol instead of isopropanol (Chunsong et al., 2015). DNA extraction kits typically finish with DNA capture on an adsorption column and elution into tubes. Generally, DNA captured by a one-pass extraction process is suitable for subsequent analysis; however, this is not the case for DNA obtained from NHP. Gao et al. successfully obtained fragments of DNA sequences from Lonicerae japonicae Flos by eluting DNA from four adsorption columns into one tube (Gao et al., 2017). Jia et al. used a specially designed extraction buffer (tris-hydrochloride: pH 8.0; 20 mM ethylenediaminetetraacetic acid: pH 8.0; 0.7 M sodium chloride; 2% polyvinylpyrrolidone-40; 4‰ β-mercaptoethanol) to remove polysaccharides, polyphenols, and pigments (Jia et al., 2017). Fatima modified the traditional CTAB extraction method by adding approximately 0.25% PVP, adjusting the concentration of RNase and proteinase, and using different incubation times (Fatima et al., 2018).

Contamination during DNA extraction is also a concern. Low-quality DNA can be easily contaminated by fungal, bacterial, or other herbal materials, which can interfere with PCR amplification (Chen et al., 2014).

## Screening and Acquiring DNA Mini-Barcodes

Typical PCR amplification of DNA barcodes is based on universal primer sets (Chen et al., 2014). However, using universal primer sets to identify all species would be difficult (Parveen et al., 2016) for several reasons. First, NHP compositions are complex and involve up to dozens of TCMs and other herbal materials. DNA barcoding is versatile for many species, but not accurate for certain species in multi-component mixtures. Moreover, the universality of barcodes can reduce the accuracy of species identification as some closely related species cannot be distinguished. Second, DNA from NHP can be severely degraded during the manufacturing process, resulting in failed amplification for standard barcodes (ITS2 > 260, *rbcL* > 500).

For the development of DNA mini-barcoding, DNA mini-barcodes must be screened and acquired. Highly specific and intra-specifically conserved fragments (100–300 bp) should be the initial standard to screen a marker for a mini-barcode. A reliable database of standard sequences should be established. These sequences could be collected from the GenBank, European Molecular Biology Laboratory, or DNA Data Bank of Japan, which have been published or uploaded by authorities. Subsequently, candidate sequences may be aligned to identify conserved and specific regions. Each mini-barcode should be proven to be unique using the Basic Local Alignment Search Tool (BLAST) analysis in standard databases with 100% identity. In addition, specific primer pairs for mini-barcode amplification should be designed for later PCR amplification. For instance, Hofreiter et al. designed primer pairs for a 157 bp amplicon in the *rbcL* region (Hofreiter et al., 2000). Lo et al. developed an adaptor primer and a target primer to identify short fragments based on the ITS2 regions of *Angelica sinensis* and *Panax notoginseng* in concentrated Chinese medicine granules (Lo and Shaw, 2018b).

Multiple sequencings in both the forward and reverse directions can be aligned and assembled into consensus contigs using software (Song et al., 2009), and low-quality fragments can be removed from the analysis. Consensus sequences should be queried in the GenBank using BLAST analysis to ensure accurate species identification. The target mini-barcode can be found in the amplified sequence.

## Major Limitations of DNA Mini-Barcodes

Although DNA mini-barcodes can help identify processed products, this technique is limited by its length constraint.

The purpose of the development of DNA mini-barcodes is not to achieve universal application for most species, but rather to identify specific targets for medicinal plants. Every PCR amplification and taxonomic result must be tested and applied. DNA mini-barcoding is unable to identify unknown adulterants or contamination, especially in complex NHP that occasionally contain >10 species. Moreover, sequences of different lengths are chosen as a mini-barcode in terms of different cases. As a result, they could not develop into standard biomarkers when dealing with all cases (Little, 2014). In addition, some important information may be missed in mini-barcodes as their length is not as long as barcoding sequences (Srirama et al., 2014).

The DNA sequence may contain unstable mutation sites or be difficult to combine into a unified sequence. Thus, selecting the position and length of DNA mini-barcodes is critical in discriminating between multiple species (Hajibabaei et al., 2006). Nucleotide signatures technique (20–50 bp in length) containing stable SNP sites could success in discrimination of closely-related species. However, this approach is useless for species that cannot be distinguished using DNA barcoding. For example, no mini-barcoding markers could be developed from ITS2 sequences to distinguish three species of *Ephedrae* herbs (*Ephedra sinica*, *Ephedra intermedia*, and *Ephedra equisetina*) as the entire ITS2 sequence fails to identify these species (Chen et al., 2019).

The development of DNA mini-barcoding should ensure reliable PCR amplification results. Although challenging, novel primer pairs based on 100–200 bp sequences must be designed that avoid dimer formation, hairpin formation, and false priming. Specially designed primer sets can drastically increase the success of amplifying degraded DNA (Hajibabaei et al., 2006).

## Comparison of DNA Mini-Barcoding and DNA Meta-Barcoding: Advantages and Disadvantages

Meta-barcoding is a technique that uses universal PCR primers to simultaneously amplify multiple DNA barcodes, identifying many species in single environmental samples (Taberlet et al., 2012). This is in contrast to DNA mini-barcoding, in which a single species is sequenced using PCR. In meta-barcoding studies on CPMs, ITS2 or other plant barcodes are frequently chosen because these sequences can produce high identification efficiency among many species. As sequencing technologies have developed, next-generation sequencing (NGS) has been widely used for NHP quality control and analysis (de Boer et al., 2017; Raclariu et al., 2017; Xin et al., 2018a,b). Most CPM prescriptions are very complex; hence, universal primer pairs for DNA barcode amplification may not be feasible for the identification of herbal products using Sanger sequencing. Identification methods combined with NGS can identify species from multiple taxa. For example, Cheng et al., inspired by Taberlet et al., used ITS2 and *trnL* (P-loop) as biomarkers to analyze nine commercial Liuwei Dihuang Wan specimens in three batches using high-throughput sequencing (HTS) (Taberlet et al., 2007; Cheng et al., 2014). Different manufacturer samples contained different contaminations, and these contaminations may have occurred during the manufacturing process. Moreover, DNA from unprocessed *Rehmannia glutinosa* was successfully amplified, whereas DNA from processed *R. glutinosa* was not. This finding indicates the different detection efficiencies of unprocessed and processed herbal materials. Coghlan et al. analyzed 26 CPMs using HTS and found unknown plant or animal sequences in 50% of samples; unexpectedly, the endangered snow leopard was detected (Coghlan et al., 2015).

Certain aspects of DNA mini-barcoding are similar to meta-barcoding. Both require shorter sequencing regions than regular barcodes (200–300 bp), and these shorter regions are amplified and sequenced. Nevertheless, differences exist between the technologies. Several preparation steps, including sequence screening and primer design are critical in mini-barcoding. The purpose of meta-barcoding is to simultaneously obtain sequences from as many species as possible. Consequently, universal primers applicable to multiple species, rather than species-specific primers, are warranted. Moreover, DNA mini-barcoding is frequently used to identify one or several related species in CPMs, while meta-barcoding is able to simultaneously identify a wide range of species or identify bulk biodiversity samples and preparations.

In meta-barcoding studies, a large quantity of sequence data must be obtained and analyzed, rendering the Sanger sequencing technique unsuitable. The next-generation Illumina sequencing system solves this problem inexpensively. However, the Illumina system is also characterized by limitations. NGS is generally accurate, especially in genome studies, while it is not the case when dealing with meta-barcoding studies of CPMs. This is because the heavily degraded DNA is unsuitable to establish a sequencing library. More PCR amplification cycles are thus warranted to increase the quality of DNA for sequencing, and the quality of sequencing reads may be substantially lower in subsequent cycles. For instance, Dohm et al. demonstrated that miscalls were distributed in guanine cytosine-rich (GC-rich) regions, and base-specific miscalls C to G or A to C were more often observed in later PCR cycles (Dohm et al., 2008). Moreover, the miscalls were caused by the inhibition of base elongation during sequencing and synthesis. Kicher et al. demonstrated declining intensities during subsequent cycles, reported increasing sequencing errors with cycle number, and found an extremely high error rate at the last base due to the incomplete phasing correction. (Kircher et al., 2009). According to a study performed by Nakamura et al., the error could be caused by lagging-strand dephasing, which can cause the sequence quality to deteriorate toward the end of reads. In addition, PCR bias toward GC-rich regions caused sequencing errors (Nakamura et al., 2011). Collectively, the aforementioned results indicate the errors inherent in NGS sequencing, which may be magnified in samples containing multiple species as cycle numbers are increased. This type of error is considered contamination, which incurs during sequencing.

In conclusion, NGS can yield large amounts of sequencing data and efficiently identify multiple species. However, sequencing errors during NGS may interfere with accurate species identification. Thus, it is necessary to align sequences along every base, allowing more accurate and reliable alignment results to be obtained. Furthermore, the analysis of unique regions of each broken barcode or mini-barcode is important. The combination of mini-barcoding and meta-barcoding has significant potential for broadening the application of barcoding for NHP identification.

## Outlook for Rapid Identification Using DNA Barcoding or Mini-Barcoding

It is occasionally necessary to rapidly detect specific ingredients in herbal medicines, and developments in DNA mini-barcoding have increased the efficiency and utility of this technique. Nucleotide signatures developed from DNA barcodes are currently considered the standard for molecular detection of certain herbal materials, avoiding sequencing. Bar-HRM analysis is based on different melting temperatures of single nucleotides. Gold nanoparticle sensors are based on a colorimetric reaction to detect single-nucleotide polymorphisms (SNP) using electrostatic adsorption. These developments are representative of the widely used technologies to detect certain species.

### Mini-Barcodes as Examples of Nucleotide Signatures

A nucleotide signature is an extremely short DNA sequence (20–50 bp), which is unique to a species and highly conserved, with 100% sequence identity through BLAST analysis. Mini-barcoding is appropriate to detect these sequences. Nucleotide signatures were initially proposed by Han’s group for identifying the TCMs Asian ginseng and American ginseng (Liu et al., 2016). Nucleotide signatures overcome the difficulties of obtaining genetic fragments from DNA-degraded and complex samples and are able to identify the target species in NHP. This technique is applicable to multiple sample types, including powders, CPMs, and extracts.

DNA mini-barcodes are shorter than a typical DNA barcode. DNA barcodes are not species-specific due to close relationships or frequent variances within a genus. To amplify a target mini-barcode, specific primer pairs replace universal primer pairs and narrow the range of species identification. Nucleotide signatures occasionally match specific primer sets and show amplification specificity. Moreover, they can also enhance the success rate and efficiency of PCR.

Nucleotide signatures have been used to authenticate several CPMs. For example, it is difficult to distinguish *Panax quinquefolium* and *Panax ginseng* in CPMs. Adulteration of these species can be distinguished using two SNP sites based on the mini-barcode sequences. In addition, the relative heights of the peaks in the sequencing results can be used to deduce the adulteration ratio (Chen et al., 2013). Based on these SNPs, a 27-bp nucleotide signature was successfully developed and implemented in 24 batches of CPMs. Detection results showed that five batches were counterfeit and two were contaminated (Liu et al., 2016). Wang et al. developed a 37-bp nucleotide signature of *Angelicae sinensis* radix. The nucleotide signature could be detected in the sequencing results of 19 CPMs labeled with *A. sinensis* radix. Different adulterants, such as *Ligusticum sinense*, *Notopterygium incisum*, *Angelica decursiva,* and *Angelica gigas,* were detected in different batches (Wang et al., 2016). A nucleotide signature for Eucommiae Folium was developed from the ITS2 region. Based on the location of the nucleotide signature, a specific primer pair was developed for Lonicerae japonicae Flos to amplify a mini-barcode containing two SNP sites targeting the adulterant Lonicerae Flos in the *Lonicera* extracts and CPMs. The results revealed the samples contained only 17 and 22% of authentic material, respectively. Two adulterants, namely Eucommiae Folium and Lonicerae Flos, were both detected in 7% of NHP in this study (Gao et al., 2017).

Thus far, nucleotide signatures for *Panax notoginseng*, *Cistanche deserticola* (Wang et al., 2018), Eucommiae Folium (Gao et al., 2017), and *Ginkgo biloba* (Liu et al., 2018b) have been developed to identify target species within NHP. Owing to its high specificity, this technique can accurately detect a species without sequencing. These studies corroborate the use of nucleotide signatures as a low-cost and efficient tool for NHP characterization.

### Bar-High-Resolution Melting Analysis for Use With Mini-Barcodes

Melting curve analysis, first introduced in the 1990s (Ririe et al., 1997), differs from mini-barcode analysis as it uses melting curves from several species and avoids sequencing. HRM analysis was developed following the advent of fluorescent dyes, and is able to detect small sequence differences with high resolution (Wittwer et al., 2003). HRM, based on PCR, precisely monitors the release of an intercalating fluorescent dye from a DNA duplex while it is denatured through a gradual temperature increase (Mishra et al., 2016). Simko briefly demonstrated the principle of this technology: double-stranded DNA used in PCR is combined with a dye (Herrmann et al., 2006; Simko, 2016). Although this dye strongly fluoresces when bound, it fluoresces at very low levels when released. After PCR, the amplicon (50–500 bp in length) is gradually denatured by increasing the temperature in small steps (0.01–0.2°C), and the fluorescent dye is slowly released from the denaturing amplicon. The diminishing fluorescence can be plotted against the increasing temperature to produce a melting curve. Factors that determine the unique shape of the melting curve include DNA strand complementarity, amplicon length, sequence, and GC content (Reed et al., 2007). Because of the sensitivity of this technology, short sequences in the genomic signature of individual species containing DNA barcodes, SNP mutations, or methylation can be inexpensively and rapidly detected using different melting curves (Wu et al., 2009; Wong and Dobrovic, 2011; Han et al., 2012; Madesis et al., 2012; Buddhachat et al., 2015; Duan et al., 2018; Mishra et al., 2018). For example, HRM is sensitive to methylation and can differentiate between methylated and unmethylated cytosine. Sodium bisulfite can convert unmethylated cytosine to uracil, while methylcytosine is unaffected by the treatment. Methylcytosine is subsequently amplified to cytosine, while uracil is amplified to thymine. This results in a lower GC content and a lower melting temperature for unmethylated sequences compared with methylated sequences (Simko, 2016).

Bar-HRM analysis combines DNA barcodes with HRM analysis and can detect genetic variants in DNA sequences. Bar-HRM analysis allows for high-throughput, rapid, and simultaneous identification of species, and it is suitable for NHP frequently adulterated with related species and genetic variants. Sequences with different variants can be clearly distinguished according to their different melting curves. Bar-HRM has been widely applied to analyze many raw herbal materials and adulterants (Kalivas et al., 2014; Buddhachat et al., 2015; Osathanunkul et al., 2015; Singtonat and Osathanunkul, 2015; Soares et al., 2018). Mishra et al. analyzed the ITS1 region using HRM to authenticate *Senna alexandrina* Mill in crude drugs. They found that *Senna* species could not be distinguished based on the 700–800-bp PCR amplicons of the ITS region. However, species-specific SNP sites and insertion–deletion mutations were detected at a total of 143 sites in the ITS region of *Senna italica subsp. italica* and *S. alexandrina*, which allowed successful simultaneous discrimination (Mishra et al., 2018).

In addition to Bar-HRM, research on herbal products with mini-Bar-HRM analysis has emerged in recent years. In one study, five medicinal *Phyllanthus* products were discriminated using Bar-HRM analysis. Two loci from *rbcL* and *trnL* were selected to generate suitable primer sets for HRM analysis specificity. Subsequently, 88- and 99-bp amplicons were produced under annealing temperatures of 50–60°C, which were the expected PCR products for the *rbcL* and *trnL* loci, respectively (Buddhachat et al., 2015).

Costa et al. developed mini-barcoding for the ITS1 and *matK* regions, coupled with HRM analysis, to authenticate *Hypericum perforatum* and *Hypericum androsaemum* in herbal infusions. Species-specific primers were developed for barcoding regions identified through PCR and real-time PCR. One primer was used to produce barcode amplicons for the regular sequencing of *Hypericum*. Another primer was designed to enable unequivocal identification through HRM analysis. When coupled with HRM, mini-barcoding of *matK* could differentiate both species. Moreover, mini-barcoding of ITS1 could identify intra-species variability, which was confirmed by HRM analysis (Costa et al., 2016).

HRM analysis has been used in many studies, indicating its value for sensitive, rapid, and cost-effective identification in combination with DNA barcoding or mini-barcoding. This technique is interchangeable with conventional post-PCR genotyping methods and can be combined with SNP sites or single sequence repeat markers to identify NHP.

### DNA-Gold Nanoparticle Sensors Combined With a Barcode Assay for Specific and Sensitive Detection

DNA-gold nanoparticle sensors have been used to rapidly detect diseases, mycotoxins, and pathogenic microorganisms (Cho and Irudayaraj, 2013; Kong et al., 2016). Gold nanoparticles (AuNPs) have a diameter of 1–100 nm, possess high electron density and dielectric properties, and can perform catalysis. AuNPs of various particle sizes can be produced by the reduction of the chloroauric acid and range from red to purple depending on their diameter. AuNPs can bind to various biomacromolecules without affecting their biological activities. Furthermore, electrostatic interactions between single-stranded DNA and AuNPs facilitate the combination of nanoparticles with DNA barcoding, as first proposed by Mirkin et al. (1996). In this technique, DNA base pairing causes the color of AuNPs to change, allowing variant sites and SNPs to be detected and analyzed.

This method involves five items: magnetic particles, AuNPs, a single-stranded probe connected to magnetic particles, a single-stranded probe connected to AuNPs, and a single-stranded target barcode sequence. Magnetic particles coated with a single-stranded probe recognize one end of the target barcode DNA, and AuNPs conjugated to a single-stranded probe combine with the other end of the target barcode DNA. This brings the magnetic particles and AuNPs together through the formation of double-stranded DNA, resulting in a magnetic particle-probe-target barcode-probe-AuNP sandwich structure (Nam et al., 2003; Rosi and Mirkin, 2005; Hill and Mirkin, 2006). The sandwich structure can be detected in several ways. This structure can be isolated in a magnetic field, and the separated barcodes can be captured using a third probe bound to AuNPs. This complex can then be identified using silver enhancement and chip-based hybridization (Park et al., 2002). In silver enhancement, nanoparticles hybridize with the oligonucleotide on chips, and are processed with silver reagent. Silver deposition is facilitated by nanoparticles, leading to readily measurable changes in conductivity and amplifying the signal detection. Alternatively, captured barcodes can be detected at nanomolar concentrations because aggregation results in a visible color change from red to blue (Verma et al., 2005; Jiang et al., 2010). Fluorescence spectrophotometry can also be used to detect barcodes (Amini et al., 2017).

AuNPs can be combined with various bioactive molecules, such as biocompatible oligonucleotides (Daniel and Astruc, 2004; Wang et al., 2010). In addition, AuNPs are easily functionalized and remarkably stable (Storhoff et al., 2000; Saha et al., 2012). Cooperative binders (Jin et al., 2003) and enhanced sensitivity (Zhang et al., 2006) further broaden the applicability of AuNPs.

Several molecular detection methods based on nanoparticles have been established to evaluate plant materials. Huang et al. developed an amplified sensor to detect DNA in genetically modified organisms using AuNPs and an electrochemical biosensor, demonstrating good sensitivity (Zhao et al., 2012; Huang et al., 2015). Lei et al. established a sequencing-free, nano-featured electrochemical DNA biosensor that was able to successfully discriminate between the two Chinese herbal species *Fritillaria thunbergii* and *Fritillaria cirrhosa* (Lei et al., 2015).

Mini-barcoding combined with AuNP sensors could be designed using a similar framework to that of biosensors used to study genetically modified organisms and detect related species. Owing to the high sensitivity of this superior signal amplification platform, mini-barcoding in combination with AuNPs could solve several problems, including unavoidable degradation in NHP, low concentrations of extracted DNA, and low PCR amplification efficiency.

**Figure 1:**
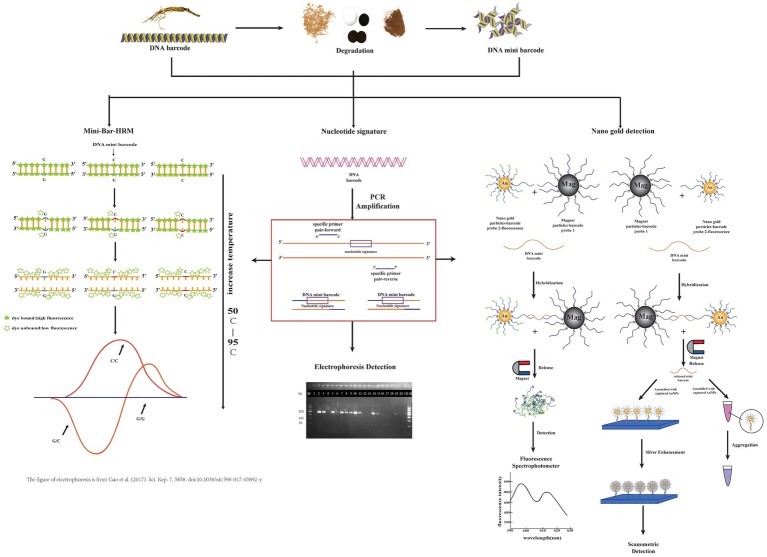
Brief procedural details of three technologies coupled with the DNA mini-barcodes involved in this review. From left to right, they are mini-bar-HRM, nucleotide signature, and nano gold detection. Some parts of this figure refer to other publications as indicated.

The experimental procedures of three key technologies are briefly illustrated in Figure 1.

## Future Expectations for DNA Mini-Barcoding

Recent media reports and scientific studies highlight how widespread adulterations and ingredient substitution in NHP have become and underscore the threat to consumer safety.

DNA mini-barcoding can overcome NHP identification difficulties caused by DNA degradation after extensive processing. While this method will never supplant typical DNA barcoding in identifying specimens, it can significantly improve the efficiency and accuracy of sample analysis by increasing the PCR amplification rate. New rapid detection methods based on mini-barcoding are expected to be established, particularly for processed products.

In general, mini-barcodes are species-specific, allowing their utility in analyzing complex samples. DNA mini-barcodes represent an improved approach to carefully identify plant products and protect public health. This significant tool should be further developed and applied to the quality control of NHP.

## Author Contributions

This review was drafted by ZG and revised by JH and YL. Other data or references were collected by XiW and XuW. All authors have read and approved the final manuscript.

### Conflict of Interest Statement

The authors declare that the research was conducted in the absence of any commercial or financial relationships that could be construed as a potential conflict of interest.

## Abbreviations

Bar-HRM: Barcoding high-resolution DNA melting
CPMs: Chinese patent medicines
CO1: Cytochrome c oxidase I
GMO: Genetically modified organism
HPLC: High-performance liquid chromatography
ITS: Internal transcribed spacer
ITS2: Internal transcribed spacer
*matK*: Maturase K
MS: Mass spectrometry
NGS: Next generation sequencing
NHP: Natural healthy products
NMR: Nuclear magnetic resonance
*psbA-trnH*: Photosystem II protein D1-tRNA-His
*rbcL*: Ribulose-1,5-bisphosphate carboxylase/oxygenase large subunit
SNP: Single-nucleotide polymorphism
TLC: Thin-layer chromatography
TGS: Third generation sequencing
TCMs: Traditional Chinese medicines.
